# Ceaseless Experimentation Sparks Fireworks, Art, and Science

**DOI:** 10.3201/eid2109.AC2109

**Published:** 2015-09

**Authors:** Byron Breedlove

**Affiliations:** Centers for Disease Control and Prevention, Atlanta, Georgia, USA

**Keywords:** art science connection, emerging infectious diseases, Emerging Infections Programs, EIP, fireworks, Edward Middleton Manigault, The Rocket, Ceaseless Experimentation Sparks Fireworks, Art, and Science, art and medicine, about the cover

**Figure:**
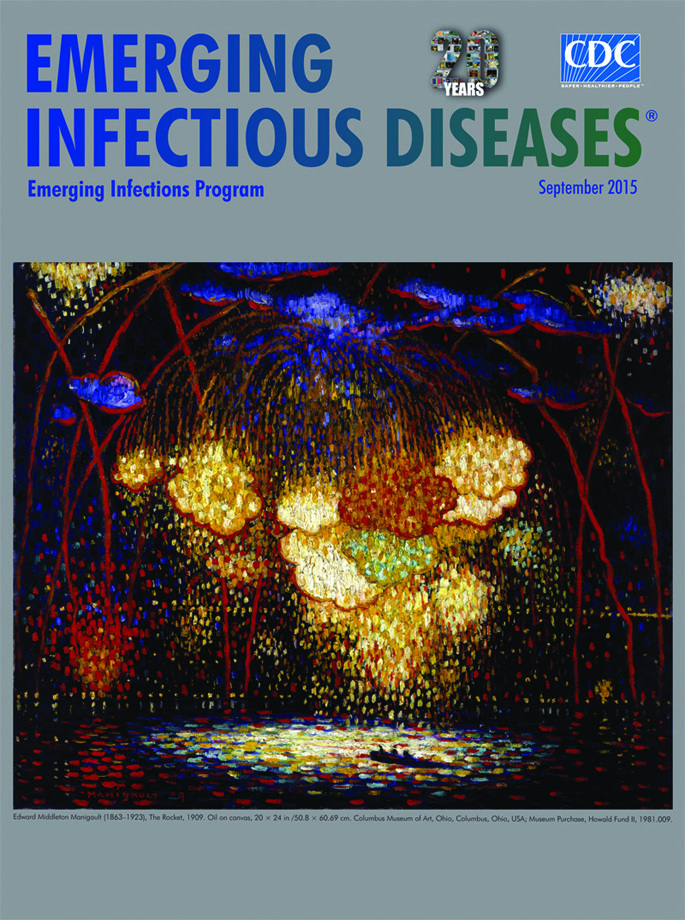
**Edward Middleton Manigault (1863–1923), The Rocket, 1909.** Oil on canvas, 20 × 24 in /50.8 × 60.69 cm. Columbus Museum of Art, Ohio, USA; Museum Purchase, Howald Fund II, 1981.009.

Fireworks and festivities have long been linked. The earliest documentation of the genesis of fireworks points to the 7th century Tang Dynasty in China. Subsequently, these celebratory practices fanned out to other cultures and countries. Some speculate that Marco Polo or returning Crusaders introduced fireworks to the West, though fireworks probably trickled in over the course of many years, tucked among the belongings of various missionaries, traders, or explorers returning from sojourns to the East. Regardless of how knowledge of fireworks seeped into Europe, once there, it took root and flourished. During the Renaissance, two European schools of pyrotechnics emerged and thrived: Italians stressed intricate fireworks displays, and Germans focused on the science behind the spectacle.

For centuries, fireworks displays were marvels of sound, fury, and smoke, as pyrotechnicians had far more success mastering the formulas for the oxidations and reductions that ensured successful ascensions and explosions than those that would consistently yield color. Experimentation during the 19th century finally yielded the formulas for mixing the various metal salts and metal oxides required to produce the brilliant colors associated with modern fireworks. A flamboyant lexicon categorizes the varieties of explosions: a peony expands outward to form a sphere of stars; fish wriggle away from the central explosion before dissolving into points of light; falling leaves are colored stars that briefly hang in the sky before drifting down slowly; kamuro are the willow-like tendrils of light that radiate out and down, a favored effect for finales. The militaristic names for the chambered vessels that streak skyward toward a brief, spectacular end are less poetic: shells, rockets, and mortars figure prominently.

Radiating and dazzling, a wonderful confluence of science, art, and skill, fireworks celebrations attract crowds around the world. This month’s cover image, “The Rocket,” by the American Modernist artist Edward Middleton Manigault, vividly portrays an evening’s fireworks show during the state of New York’s 1909 Hudson-Fulton Celebration. This civic event commemorated the 300th anniversary of Henry Hudson's discovery of the river now bearing his last name and the 100th anniversary of Robert Fulton's inauguration of steamboat travel on the Hudson River (although the actual centennial was in 1907).

“The Rocket” bristles with energy; it is a dramatic, imaginative, and colorful reckoning of the celebration. Fireworks streak into the night sky, their explosions rendered by bold red, orange, and yellows brush strokes dabbled against the black night and blue clouds. Glowing ribbons of red light streak skyward and rim the blue clouds and the glowing fireworks clustered in the center of the painting, adding structure and symmetry like the lead cames in a stained glass window. The water refracts and reflects the spectacle, as a lone small boat sits on the river, nearly overwhelmed by the radiant display showering down. Myriad reflected splotches of color coat the surface of the river, like a vividly colored array of water lilies.

Manigault relied on Impressionistic style and technique in creating “The Rocket,” during what is considered the peak of his career. Manigault restlessly and persistently explored new styles and influences in his personal quest to fathom the power and intensity of color. He would focus intently on specific styles and methods for several years and then shift to mastering a new approach—one of his admirers has dubbed Manigault’s artistic focus as “ceaseless experimentation.”

The notion of “ceaseless experimentation” also resonates well within the sciences, where the collaborative power of networks allows programs the chance to investigate and broadly test and evaluate ideas and approaches. It has been 20 years since the Centers for Disease Control and Prevention launched the Emerging Infections Program. This national resource for surveillance, prevention, and control of emerging infectious diseases expands the routine activities of participating state health departments by joining with academic partners and strives to translate surveillance and research activities into public health policy and practice.

The Emerging Infections Program—which debuted in 1995—traces its genesis in part to the Institute of Medicine’s 1992 report *Emerging Infections: Microbial Threats to Health in the United States* and more specifically to the Centers for Disease Control and Prevention’s 1994 report *Addressing Emerging Infectious Disease Threats: a Prevention Strategy for the United States*. This special-themed issue of the *Emerging Infectious Diseases* journal offers a wide-ranging perspective of the Emerging Infections Program’s activities and accomplishments, and Manigault’s dynamic painting helps us celebrate those two decades of research, collaboration, and publication with the appropriate flare.
